# Employee Physical Activity in an Outpatient Oncology Clinic: A Baseline Pilot Study

**DOI:** 10.7759/cureus.3803

**Published:** 2018-12-31

**Authors:** Valerie Reed, Anita Kaw, Hiral Patel, Isidora Arzu, Lauren Mayo, Lorenzo Cohen, David Rosenthal, Shalin Shah

**Affiliations:** 1 Radiation Oncology, The University of Texas MD Anderson Cancer Center, Houston, USA; 2 Integrative Medicine, The University of Texas MD Anderson Cancer Center, Houston, USA

**Keywords:** employee health, employee physical activity, pedometer, employee wellness

## Abstract

Purpose: Studies examining the physical activity of employees within an outpatient oncology setting are absent. The goal of this pilot study was to collect baseline data on the daily activity of employees in varying job descriptions at a satellite outpatient oncology clinic of a large academic medical center.

Methods: A total of 40 employees (out of a total of 55) were accrued on this clinical trial. Each employee was given a pedometer to wear at work for a total of 20 business days, with instructions not to alter their baseline activities. Employees recorded their daily workplace pedometer activity on a personalized chart. Baseline vital signs, as well as their general job title, were recorded.

Results: Of the 40, 36 employees (90%) completed the study. The average steps per workday for all employees were 4455 +/- 2051, which is significantly less than the recommended 10,000 steps per day (p <0.001). There was a statistically significant difference in activity between various job descriptions, with radiation therapists having the highest daily mean steps (8853 +/- 930) and front desk staff having the lowest mean steps (3147 +/- 1010), p<0.001).

Conclusion: Employees at a satellite outpatient oncology clinic of a large academic center, on average, do not meet the surgeon general’s recommendations for daily physical activity at the workplace, with only radiation therapists approaching the recommended steps.

## Introduction

The United States Department of Health and Human Services designated physical activity as the leading health indicator for Healthy People 2020. The United States Center for Disease Control (CDC) reports that adults should perform 2.5 hours of moderate-intensity activity per week [1] while the World Health Organization (WHO) recommends that people should perform at least 30 minutes of moderate-intensity physical activity on most days, as it reduces the risk of cardiovascular disease, diabetes, and some cancers [2]. Although the health benefits of physical activity are recognized, less than 50% of American adults are meeting the current activity guidelines [3]. This phenomenon is likely to continue, as physical activity is being reduced in all aspects of the life environment, including home, work, recreation, and transportation.

An individual’s physical activity is strongly linked to the worksite. The workplace is recognized as a priority setting for health promotion by the World Health Organization [4]. A shift towards more sedentary jobs has led to a decline in workplace physical activity and closely mirrors the nation’s weight gain curves over the past 50 years [5].

Employers are becoming more interested in employee physical activity, as healthcare costs are a significant and growing aspect of employment costs. One study showed that obesity alone was estimated to cost almost $2,500 per employee per year, including direct medical costs and missed work days [6]. Additionally, a 1% reduction in excess weight and high blood pressure, glucose, and cholesterol levels has been shown to save $83 to $103 annually in medical costs per person [7].

Pedometers are frequently used in workplace health promotion programs. A pedometer is a small, portable electronic device that counts the number of steps by an individual. These programs encourage employees to wear the pedometer to record activity and often provide a target step goal for participants such as the commonly used 10,000 steps per day [8-9]. Studies examining the physical activity of employees within an outpatient oncology setting are currently lacking. Thus, the goal of this pilot study was to collect baseline data on the daily activity of employees of varying job descriptions at a satellite outpatient clinic of a large academic medical center.

## Materials and methods

Recruitment

All study procedures were submitted to and approved by the Institutional Review Board prior to beginning any research activities. All participants provided written informed consent. The study population consisted of employees at a suburban outpatient oncology satellite clinic of a large academic medical center (X Regional Care Center). Employees who were unable to walk due to disabilities were excluded from the study. A total of 40 employees (of 55 total employees) were enrolled in the study. Their general job title was recorded.

Intervention

Participants were given a step-counting pedometer (Fitbit Zip, Fitbit Inc., San Francisco). Employees were instructed to wear the pedometer for a period of 20 business days (one month) during the workday. Instructions not to alter their baseline activity were provided. 

Outcome measures

Outcome measures included pedometer step counts as an estimate of daily physical activity.

Statistics/data analysis

To test whether the global sample mean differed significantly from the surgeon general’s recommended 10,000 steps per day, a one-sample t-test was used (Microsoft Excel) was used.

To test the difference in mean steps between the various groups (arranged by job descriptions), a single factor analysis of variance (ANOVA) was employed (Microsoft Excel). Further, a post hoc, single-step multiple comparison (i.e., Tukey’s) was not performed due to the limited sample size (and relatively large standard deviation (SD)) in some groups.

## Results

Of the 40 employees, 36 (90%) completed the study. Employees with various job titles participated and completed the study (Table 1). Front desk/business center staff comprised the majority of participants (10/36, 28%), followed by non-infusion nurses (7/36, 19%), infusion nurses (6/36, 17%), nurse assistants (6/36, 17%), radiation therapists (3/36, 8%), radiation physicists (2/36, 5%), and administrative assistants (2/36, 5%).

**Table 1:** Employee step count by job title

Employee Title	N (%)	Mean step count (SD)
Front desk/business center	10 (28%)	3136.5 (1010.9)
Non-infusion nurse	7 (19%)	3893.3 (881.5)
Infusion nurse	6 (17%)	5154.4 (583.7)
Nurse assistant	6 (17%)	4813.8 (2734.2)
Radiation therapist	3 (8%)	8853.3 (934.9)
Radiation physicist	2 (5%)	3387 (2674)
Administrative assistant	2 (5%)	4292.3 (773.8)
Total	36	4455 (2051)

The mean step count per workday for all employees was 4455 +/- 2051. There was a significant difference in activity between various job descriptions (Table 1), with radiation therapists having the highest daily mean step count (8853 +/- 930) and front desk/business center staff having the lowest mean steps (3147 +/- 1010), p<0.001.

## Discussion

Although studies describing the results of pedometer-based interventions are fairly common in the medical literature, there are very few, if any, studies examining the physical activity of employees within an outpatient oncology setting.

This study demonstrated that the employees of a satellite outpatient oncology clinic of a large academic medical center do not, on average, meet the 10,000 steps/day goal. The three radiation therapists approached this goal the closest, with 8853 mean steps/day.

The limitations of the study include low sample size, which limits the data analysis, though the majority of employees did participate in the study. Furthermore, a pedometer is not able to quantify non-ambulatory activities. Another limitation of this study is that it only studied step count at the workplace, not outside of work, where employees could have been more active. However, a growing body of evidence also suggests that exercise after work cannot compensate for inactivity during work, given the time differential between the two. One study demonstrated that inactivity in the workplace is an independent risk factor for cardiac death, regardless of whether exercise is undertaken after work hours [10].

Given the strong associations between physical inactivity and cardiovascular risk, workplace programs striving to increase the overall employee physical activity have the potential to improve the cardiovascular health of many employees. Encouraging employees to take brisk walks during breaks and to stand more frequently during the workday added 6-10 minutes of walking each day, [11] which equals approximately 600-1000 additional steps/day [12].

## Conclusions

In summary, this study provides a baseline assessment of workplace activity for employees at an outpatient oncology clinic. On average, employees did not meet the 10,000 steps/day goal. This baseline study will be useful for designing worksite wellness programs in outpatient oncology clinics. Worksite wellness programs that improve physical activity have the potential to reduce the physical and economic burden of diseases associated with low physical activity for diseases associated with low physical activity. Future studies should explore worksite wellness activities as well as the financial impact of decreased activity in the workplace.

## References

[1] (2018). How much physical activity do adults need?. cited.

[2] (2018). WHO. Global strategy on diet, physical activity and health. http://www.who.int/dietphysicalactivity/strategy/eb11344/strategy_english_web.pdf.

[3] (2018). National Center for Health Statistics. Exercise or physical activity. cited.

[4] Organization WH. Workers (2018). WHO Global Plan of Action on
Workers’ Health (2008-2017):. Sixtieth World Health Assembly. Geneva: WHO.

[5] Tudor-Locke C, Bassett DR (2004). How many steps/day are enough? Preliminary pedometer indices for public health. Sports Med.

[6] Finkelstein E, Fiebelkorn lC, Wang G (2005). The costs of obesity among full-time employees. Am J Health Promot.

[7] (2018). At A Glance 2015 Workplace Health Promotion. At A Glance 2015 Workplace Health Promotion.

[8] Behrens TK, Domina L, Fletcher GM (2007). Evaluation of an employer-sponsored pedometer-based physical activity program. Percept Mot Skills.

[9] Dishman RK, DeJoy DM, Wilson MG, Vandenberg RJ (2009). Move to Improve: a randomized workplace trial to increase physical activity. Am J Prev Med.

[10] Tudor-Locke C, Leonardi C, Johnson WD, Katzmarzyk PT (2011). Time spent in physical activity and sedentary behaviors on the working day: the American time use survey. J Occup Environ Med.

[11] Gilson ND, Puig-Ribera A, McKenna J, Brown WJ, Burton NW, Cooke CB (2009). Do walking strategies to increase physical activity reduce reported sitting in workplaces: a randomized control trial. Int J Behav Nutr Phys Act.

[12] Marshall SJ, Levy SS, Tudor-Locke CE (2009). Translating physical activity recommendations into a pedometer-based step goal: 3000 steps in 30 minutes. Am J Prev Med.

